# Forecasting the future of American mass shootings with Bayesian agent-based model calibration

**DOI:** 10.1093/pnasnexus/pgag294

**Published:** 2026-09-22

**Authors:** Andrew M Dickson, Mira Bhatt, Tiffany Yu, Mohammad R K Mofrad

**Affiliations:** Departments of Bioengineering and Mechanical Engineering, University of California, Berkeley, CA 94720, USA; Departments of Bioengineering and Mechanical Engineering, University of California, Berkeley, CA 94720, USA; Departments of Bioengineering and Mechanical Engineering, University of California, Berkeley, CA 94720, USA; Departments of Bioengineering and Mechanical Engineering, University of California, Berkeley, CA 94720, USA; Molecular Biophysics and Integrated Bioimaging Division, Lawrence Berkeley National Laboratory, Berkeley, CA 94720, USA

**Keywords:** agent-based modeling, Bayesian analysis, mass shootings

## Abstract

Mass shootings represent a persistent and devastating public health crisis in the United States, yet their rarity makes them difficult to model using standard statistical approaches. This is especially true for agent-based models (ABMs), which offer a mechanistic framework for understanding human behavior, but have historically struggled to capture low-probability, high-impact events in a way that is both interpretable and statistically well-calibrated. Here, we develop a high-throughput ABM that models mass shootings as arising from the intersection of three factors: the emergence of a motivated potential perpetrator, access to firearms, and the presence of a suitable target population. Through density estimation of the predicted severity of events from our ABM model, we evaluate the log-likelihood of historical US mass shooting data, enabling Bayesian posterior inference over the ABM parameters. We then apply variational Bayesian Monte Carlo (VBMC) methods to fit ABMs to historical data, while also producing predictive distributions with principled uncertainty quantification. Calibrated models are directly compared through Bayesian metrics and yield estimates of latent societal parameters, such as the baseline rate at which potential perpetrators emerge, that are grounded in a plausible generative process. We find that Federal Firearms License density is the dominant predictor of risk while population density has a positive but quickly saturating, impact. Through Widely Applicable Information Criterion evaluation of models, we find that an ABM accounting for high-lethality weapons, but assuming randomized mass-shooting targets, outperforms both naive Poisson–Weibull baselines and plausible behavioral variants. Our results demonstrate that mechanistic ABMs calibrated by VBMC can match or exceed standard statistical baselines while supporting counterfactual policy simulation.

Significance Statement Using a novel framework for the training and evaluation of agent-based model (ABM) ensembles, we show that ABMs conditioned on coarse demographic information can meaningfully predict where mass shootings are likely to occur and outperform standard statistical baselines. Our findings specifically highlight local firearm availability, proxied by Federal Firearms License density, as a significant risk factor, and predict the spread, and severity of potential future mass shootings.

## Introduction

Mass shootings have become a persistent public health crisis in the United States. The increasing frequency and severity of these events pushes an urgent need for comprehensive research and policy-driven interventions. Unlike other forms of gun violence, mass shootings are relatively rare but disproportionately impactful, creating widespread fear and prompting legislative debates (1, 2). Yet from a scientific perspective, mass shootings present a challenging modeling problem: they are extremely rare, and shaped by complex interactions between individuals and their local environment.

One promising avenue is the use of policy simulation and modeling for risk assessment and policy outcomes (3). With simulation techniques, policymakers can prioritize strategies with the greatest impact on reducing mass shootings. However, typical modeling approaches face significant challenges when applied to mass shootings due to their inherent rarity and unpredictability (4). Under reasonable inclusion criteria, fewer than 160 recorded incidents exist in the US dataset (5); a sample size of this scale allows for the calculation Poisson-rate constants (4), but more complex, outlier-style statistical models in this regime have limited ability to generalize or provide robust confidence estimates of more complex models (6).

This said, mass shootings are a human phenomena, dependent on social dynamics, and relatively complex agent-based models (ABMs) are an attractive option for modeling such situations. ABMs efficiently describe individual interactions giving rise to aggregate patterns (7). They are widely used in fields such as sociology, economics, and biology to model phenomena ranging from housing discrimination to the spread of COVID-19 (8, 9). In the context of mass shootings, ABMs can encode hypotheses about how potential perpetrators emerge, how access to firearms occurs, and how population structure affects target availability. They already show some success in modeling social outcomes in scenarios such as the impact of public policy decisions on violence per capita (10, 11).

However, ABM models are particularly vulnerable to data sparsity, which constrains their use for prediction and policy applications. With only 160 data points over four decades (5), most ABMs flexible enough to recapitulate mass shooting events are likely to be only semidetermined. For them to generate plausible, interpretable predictions, ABMs applied to mass shootings should be calibrated to match real-world data, and come with confidence bounds on predictions and critical parameters. Because their dynamics are typically nonanalytic and nondifferentiable, this represents a computationally demanding, and often subjective, task (12, 13).

In this work, we address these challenges by embedding an ABM of mass shootings within a Bayesian inference framework. We treat mass shootings as the outcome of three interacting stochastic processes: the emergence of a motivated perpetrator, the acquisition of a firearm, and the selection of a target population. Firearm access and population density are relatively well-characterized inputs that prior empirical work links to community gun violence, while perpetrator emergence is essentially impossible to estimate directly from public data. Our framework is designed precisely to test the space of plausible behavioral assumptions about emergence against these better-constrained inputs. Geographic firearm availability, whether proxied by state-level gun-ownership rates or by the density of licensed firearm dealerships, has been linked specifically to the frequency and severity of mass shootings (14, 15). Population density is a similarly recurrent correlate, predicting both county-level mass-shooting incidence (16) and broader spatial patterns of homicide (17). Both quantities are also available at high spatial resolution across the entire country, making them practical drivers for a nationwide mechanistic simulation directly targeted at mass shootings.

In our work, we ensemble thousands of iterations of simulation in order to estimate the log-likelihood of historical events under parametrized ABM predictions. The outcomes of mass shootings are represented through a single variable, the number of fatalities resulting from the incident, making it possible to directly estimate the probability density function of ABM outcomes instead of relying on pseudolikelihoods or surrogate modeling (18, 19).

We apply Variational Bayesian Monte Carlo (VBMC) to recover full posterior distributions over ABM parameters. This approach enables statistically principled comparison of alternative mechanistic hypotheses, while preserving the interpretability of ABM parameters as real-world quantities such as firearm access or population risk (20).

Using demographic data from the US Census and firearm access data from the Bureau of Alcohol, Tobacco, Firearms, and Explosives, we show that calibrated ABMs can meaningfully reproduce observed spatial patterns of mass shooting risk. Our results identify local firearm availability, proxied by Federal Firearms License (FFL) density, as a key predictor of event frequency and severity, while population density exerts only a limited effect.

More broadly, under the assumption of a particular ABM framework, we are able to infer latent parameters such as the per-capita rate of perpetrator emergence or the effective radius of firearm access, directly from historical data, and directly compare plausible competing models for mass shooting related violence. In our overall work, we test whether demographic inputs (population density and local firearm-license density) carry sufficient signal to predict the spatial distribution of mass-shooting events at the subcounty level when embedded in a mechanistic ABM. We then evaluate whether mechanistically different ABM variants, which account for high-lethality weapon access or targeted shooter behavior, fit the observed severity distribution better than variants that do not. Each hypothesis is evaluated through Bayesian posterior inference and Widely Applicable Information Criterion (WAIC)-based model comparison rather than by point estimation alone, allowing for direct comparison with statistical baselines and principled uncertainty quantification.

## Materials and methods

### Model description

**Figure 1:**
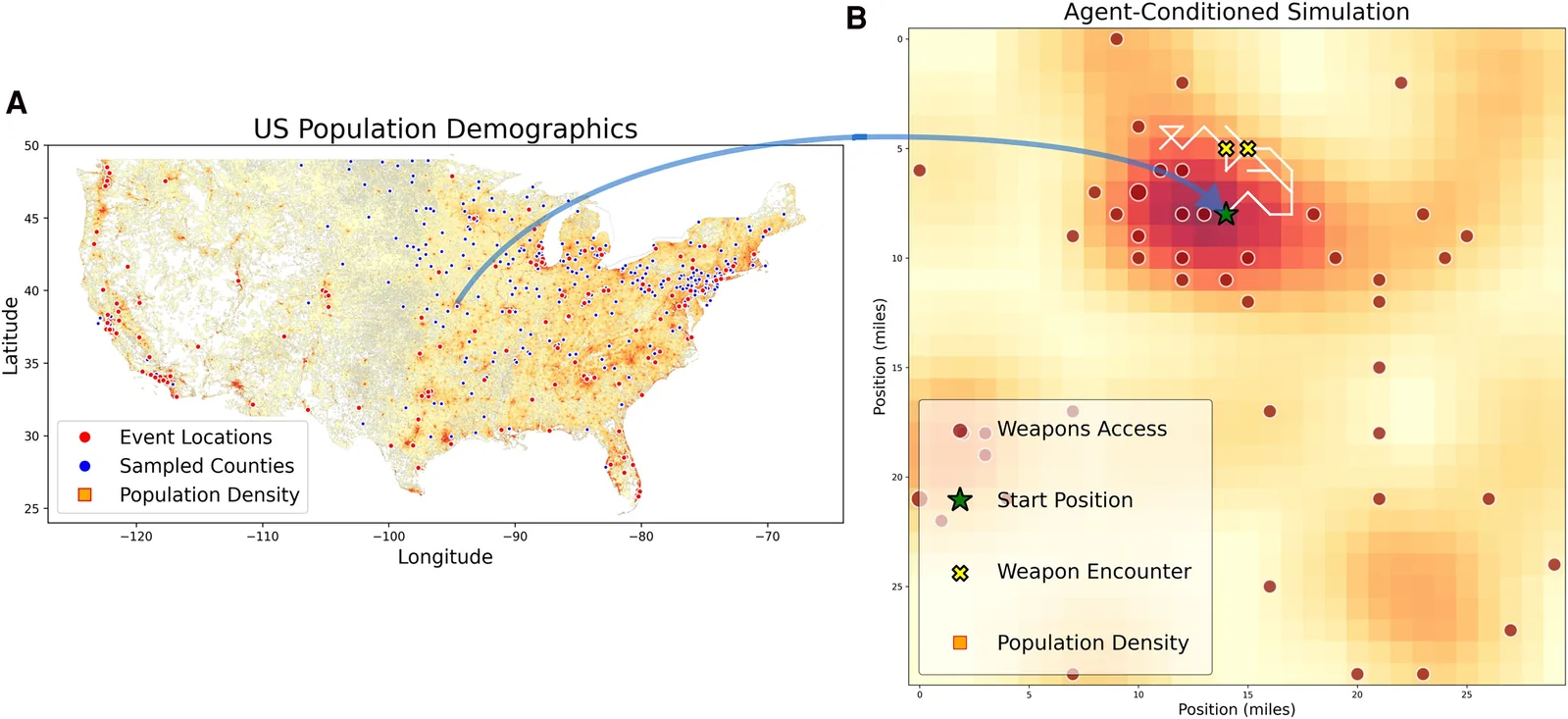
High-level description of the agent-based modeling framework for American mass shootings. A) Each location in America, as represented by NHGIS subcounty data, is associated with demographic data, and potentially sampled as context for an ABM simulation. All locations in which mass shootings occurred, and a subset of additional American counties, are sampled as datapoints for simulation. B) ABMs conditioned on the location data and global model parameters may then model the potential occurrence of a mass shooting. The ABM models agent behavior as a random walk on grid cells representing square miles of local area. Grid cells are associated with a local population density and zero or more firearms access points, which are respectively used to model the number of at-risk locations, and the potential access to firearms, for the agent. A single ABM run outputs scalar severity estimates corresponding to the number of fatalities potentially resulting from a mass shooting.

We consider a simplified model of mass shooting events in America in which mass shootings are treated as the result of a confluence of a motivated agent, acquisition of a firearm, and the choice of a target (Fig. 1). Mass shooting context is considered to be defined by the US subcounty (NHGIS Minor Civil Division or Census County Division), to which each event from the Mother Jones dataset is assigned via its recorded latitude–longitude coordinates and great-circle distance to the nearest subcounty centroid (see Materials and methods). Because of the extreme rarity of motivated agents (21, 22), we model their occurrence in a given location as a Poisson distribution, with parameter proportional to the location population by a per-person propensity factor $\lambda_{\mathrm{prop}}$ assumed to be constant across time periods and geographic location.

Conditional on the existence of a motivated agent and their geographic location, we use an ABM to simulate their acquisition of a weapon and potential targets, and the eventual severity of a potential mass shooting. ABM simulations are conditioned on the population density (people/sq mile) and firearm acquisition site density (locations/sq mile) of the local area. In addition, ABM models are parametrized by four parameters representing the distance covered by an agent within the simulation, the baseline rate of appearance of firearms acquisition sites per square mile within the simulation, the increase in firearms acquisition sites per additional firearm license per square mile, and the rate of increase of at-risk locations with population density. An augmented simulation uses an additional unitless mixture rate parameter to predict the ratio of differing agent behaviors, for a total of five to six parameters for each complete model.

The local geographic area of an agent is simulated as a 30×30 grid, with cells representing 1 sq mile of space. The 30-mile cell extent was chosen as a plausible upper bound on the travel radius of a single agent. We additionally evaluate calibration and predictive performance across alternative grid resolutions in SI Appendix, Section S3; conclusions are stable across the range of plausible grid sizes. Grid cells are associated with a population density, with the maximum population density determined by historical records (23), and heterogeneity introduced through a Gaussian mixture model that produces realistic gradients between densely and sparsely populated areas.

Because the only outcome reported by our ABM simulations is the per-event severity, defined as the number of fatalities resulting from a single mass-shooting event, we may estimate the distribution defined implicitly by a specific parametrization through density estimation methods. We apply the Grenander estimator (24, 25) to evaluate the probability density function of event severities from ensembles of 50,000 ABM simulation outcomes (see SI Appendix, Section S2.1). For efficiency, ABM simulations are implemented in Jax (26), and run in parallel on GPU.

### Data collection

We compiled a dataset of mass shooting events in the United States spanning 1982–2024, from the Mother Jones mass shooting database (5, 27). Incidents were required to involve three or more fatalities, excluding the perpetrator, and were limited to nonterrorism, nongang, and nondomestic-dispute cases (28). Each incident was treated as a point event in space and time and assigned to a US county subdivision (Minor Civil Division or Census County Division; hereafter “subcounty”) for analysis.

Subcounty boundaries, population counts, and land area were obtained from the National Historical Geographic Information System (NHGIS) decennial census files (23). For each shooting, demographic covariates were taken from the nearest decennial census to the event year (e.g. 2010 data for events in 2005–2014). Population density was computed as the total population divided by the NHGIS-reported land area of the subcounty. To map events to subcounties, each shooting latitude–longitude was linked to the nearest subcounty centroid using great-circle distance.

When a subcounty experienced multiple shootings within the same decade, each incident was retained as a separate observation. We structured the dataset in subcounty-by-decade units, generating a panel in which all subcounties contribute data for each decade within the Mother Jones data collection period, including those with zero events.

To quantify local access to firearms, we incorporated data on Federal Firearms Licenses (FFLs), drawing on the Bureau of Alcohol, Tobacco, Firearms and Explosives (ATF) listings for 2025 (29). We used the density of Type 01 (dealer), Type 02 (pawnbroker), and Type 07 (manufacturer) licenses as a proxy for local firearm availability (1, 30, 31), acknowledging that contemporary FFL distributions do not reflect historical access but may capture structural spatial patterns persisting across decades.

For each subcounty, we computed the number of FFL licensees within a 15-mile radius of its centroid and converted this to a density by dividing by the area of the circular buffer. This yielded a spatially smoothed, localized measure of firearm marketplace intensity. No additional socioeconomic or demographic covariates were included.

### Downsampling and weighting

Each data point in our analysis corresponds to a single mass shooting event from the Mother Jones mass shooting database, with associated demographic and contextual variables defined at the subcounty level. The full dataset contained ≈200,000 subcounty level observations, but running the model on all entries was computationally infeasible. With ≈50,000 ABM model simulations per county and roughly 50,000 counties nationwide, each model evaluation would require over an hour of runtime.

To make the model computationally feasible while maintaining a representative sample of the data, we downsampled those datapoints without corresponding events with probabilities monotonically increasing with each of the three variables used as input. We then upweighted subcounty datapoints by the inverse of their sampling probability to avoid biasing log-likelihood evaluations. Through manual checks, we confirmed that our subsampled data included samples from all strata of input variables in our dataset. Subcounties were sampled independently according to these probabilities, generating a target sample size of 500 datapoints.

### Statistical modeling

Our modeling framework links a statistical model of perpetrator emergence with a conditional ABM that simulates the severity of individual mass shooting events. The model begins with a generative assumption that each subcounty *i* may, within an observation window *T*, produce zero or more motivated individuals who attempt a mass shooting. We treat these occurrences as a Poisson process, where the expected number of motivated agents $\mu_{i}$ is proportional to the subcounty’s population $c_{i,\text{pop}}$, the time interval *T*, and a per-capita appearance rate $\lambda_{\mathrm{prop}}$:


(1) $$N_{i}∼\text{Poisson}(\mu_{i}),\;\mu_{i}=\lambda_{\mathrm{prop}}\;c_{i,\text{pop}}\;T.$$

Conditional on the existence of a motivated agent, and demographic factors of local population density and spatial access to firearms, we may then predict the distribution of severities of a potential mass shooting from the potential outcomes of our ABM. The ABM represents each subcounty as a 30×30 grid of one–square-mile cells, with local population density assigned to cells according to a Gaussian mixture model designed to match typical population heterogeneity (see SI Appendix, Section S2.5). Zero or more firearm access points (“FFL locations”) appear in each cell according to a Poisson process with local rates proportional to population density, and total rate for the simulation per square mile of $\lambda_{\mathrm{FFL}}=\alpha +\beta d_{i}$, where $d_{i}$ is the local FFL density and *α*, *β* are learned parameters controlling baseline and density-dependent access.

Zero or more at-risk locations appear in grid cells, according to a Poisson process with rate proportional to population density. Agents are assumed to have a baseline expected value of one at-risk location. Agents perform a bounded random walk through the grid, and an event occurs if they encounter both an access point and an at-risk population cluster. Nonevents are recorded as outcomes with severity zero by the model.

Event severity is modeled as a truncated exponential random variable, capped at the number of individuals in the affected gathering. The resulting severity distribution is monotonically decreasing, and its probability density function is estimated nonparametrically using the Grenander estimator (see SI Appendix, Section S2.1). In simulations modeling weapon category, the exponential decay rate is set according to type: (e.g. pistols) use a steeper rate corresponding to fewer expected fatalities, while high fatality events (e.g. semiautomatic rifles) use a flatter rate, producing heavier tails. Gathering size for upper truncation is sampled from a Weibull distribution tuned to match the empirically observed distribution of human gathering sizes (32), and in the case for max-select agents, set to the maximum of samples for each at-risk population encountered within simulation. The Weibull scale parameter is set to match the lower envelope of empirically observed gathering-size statistics (32); values substantially below this regime were not treated as plausible alternatives. The accompanying sensitivity analysis (SI Appendix Section S3) tests scale values within the empirically supported range.

Combining the Poisson appearance model with this conditional severity distribution yields a complete likelihood for the observed dataset D, consisting of all subcounties and their recorded events. In order to account for recording bias, we define $p_{c_{i}\geq 3}=\int_{3}^{\infty }f_{\theta_{city}}(x|\theta_{\mathrm{general}})dx$ to represent the probability of the shooting event ensuing from a motivated agent qualifying as a mass shooting and being recorded. The likelihood of our recorded dataset is then:


(2) $$\begin{array}{rl} P(D|\theta_{\mathrm{general}}) & =\prod_{i}[\frac{(\mu_{i}p_{c_{i}\geq 3}{)}^{N_{i}}e^{-(\mu_{i}p_{c_{i}\geq 3})}}{N_{i}!}\\ & \;\prod_{\;j=1}^{N_{i}}\frac{1}{p_{c_{i}\geq 3}}f_{\theta_{i}}(x_{ij}|\theta_{\mathrm{general}})], \end{array}$$

where $f_{\theta_{i}}(x_{ij}|\theta_{\mathrm{general}})$ is the ABM-derived probability density of observing an event of severity $x_{ij}$ in subcounty *i*. The likelihood simultaneously accounts for both the frequency and magnitude of events.

Model parameters $\theta_{\mathrm{general}}$ are estimated via Bayesian inference, in which posterior distributions are approximated using VBMC, which constructs a Gaussian-mixture surrogate of the posterior surface based on repeated ABM likelihood evaluations. For each ABM model, we run VBMC until convergence, in practice requiring 600–1,200 dataset log-likelihood evaluations per model. All results are generated using the PyVBMC package.

Model selection is performed using the Widely Applicable Information Criterion (WAIC) computed from posterior log-likelihood samples (33, 34). WAIC metrics were estimated from 500 Monte Carlo samples of the posterior log-likelihood for each model. Because the dataset included weighted subcounty observations, a custom implementation of the WAIC calculation was used to correctly account for sample weights in the effective number of parameters and variance estimates.

#### Statistical baseline

A simple baseline for gun violence prediction is a hybrid event-severity statistical model, as applied in prior work in modeling mass shootings as outlier events (4, 6).

Here, we model the occurrence of an event as Poisson distributed, with parameter proportional to population: $N_{i}∼\text{Poisson}(\mu_{i})$, $\mu_{i}=\lambda_{\mathrm{prop}}\;c_{i,\text{pop}}$. Conditional on event occurrence, we then model shooting severity as a two parameter Weibull distribution in order to account for heavy-tailed events. Bayesian posteriors over the event-severity model are estimated using the NUTs sampler in the PyMC package (35), using the same weighted dataset as for all ABM models.

### Model comparison

Each ABM model represents a potential mechanistic model of mass shooting scenarios. A critical feature of Bayesian modeling of ABM parameters is that it allows us to compare several mechanistically different models on the same statistical basis. We develop three models from the same basic premise of an agent acquiring a weapon and selecting a target, with different implications for the behavior of motivated agents.

Our control model is one in which weapon access is modeled as a binary outcome, in which contact with any grid cell containing one or more access points implies access. We then assume that if the agent comes into contact with one or more at-risk locations, they will uniformly randomly select one of these locations as a target, with a randomly distributed number of people present (see SI Appendix, Section S2.1). The severity of an event is then modeled as a low-discount factor exponential with a maximum severity of the number of people at the at-risk location.

Records of mass shootings show that an appreciable fraction involve more dangerous weapons, such as semiautomatic rifles. To account for this, we develop an augmented model in which access to higher weapons is accounted for with an additional binary variable. We model each encounter with a weapon as providing a one in ten chance of high-weapon access. We adopt the 1/10 per-encounter probability as a tractable approximation calibrated to the empirical pistol-to-semiautomatic-rifle ratio in our mass shooting database (27); it is not directly measured at the encounter level. The sensitivity of posterior estimates and predictive performance to this value is reported in SI Appendix, Section S3. We then model event severity as either a low-mean truncated exponential with access to only basic weapons, or a high-mean exponential with access to higher. This effectively caps the highest possible number of fatalities in a low-weapon event as on the order of 15.

Motive and intention also play critical roles in the outcomes of mass shooting events. We test one possible model of intention by extending our model to assign a percentage chance for the agent to select the at-risk location with the largest gathering size, and the highest potential for casualties. Such agents are termed as “max-select,” and account for the possibility of mass shootings with the primary motive of fatalities. We may then rigorously evaluate the fit of this model against the default model, which assumes random target selection, to test whether casualty-maximization is a plausible default behavioral profile for mass shooters.

## Results

We evaluate three structurally distinct ABM frameworks against a simple non-ABM statistical baseline, all calibrated on the same subcounty dataset and compared on the same WAIC scale (see Methods, “Model Comparison”). The three ABM variants share a common generative premise in which a motivated agent emerges, acquires a firearm if they encounter a marketplace, and selects a target if they encounter an at-risk location. They differ in how weapon lethality and target selection are modeled. The Baseline ABM treats firearm access as binary and target selection as uniform-random over encountered at-risk locations, with severity drawn from a single truncated exponential capped at the gathering size. The High/Low Arm variant adds a 1/10 probability of high-lethality access at each marketplace encounter, with severity drawn from one of two truncated exponentials according to access type. The High/Low Arm + Max-Select variant additionally introduces a behavioral-mixture parameter assigning a probability that the agent selects the highest-casualty location encountered, rather than choosing uniformly at random. The non-ABM Baseline Naive comparator is a hybrid Poisson–Weibull model with no demographic conditioning, included as a reference (4, 6).

**Figure 2:**
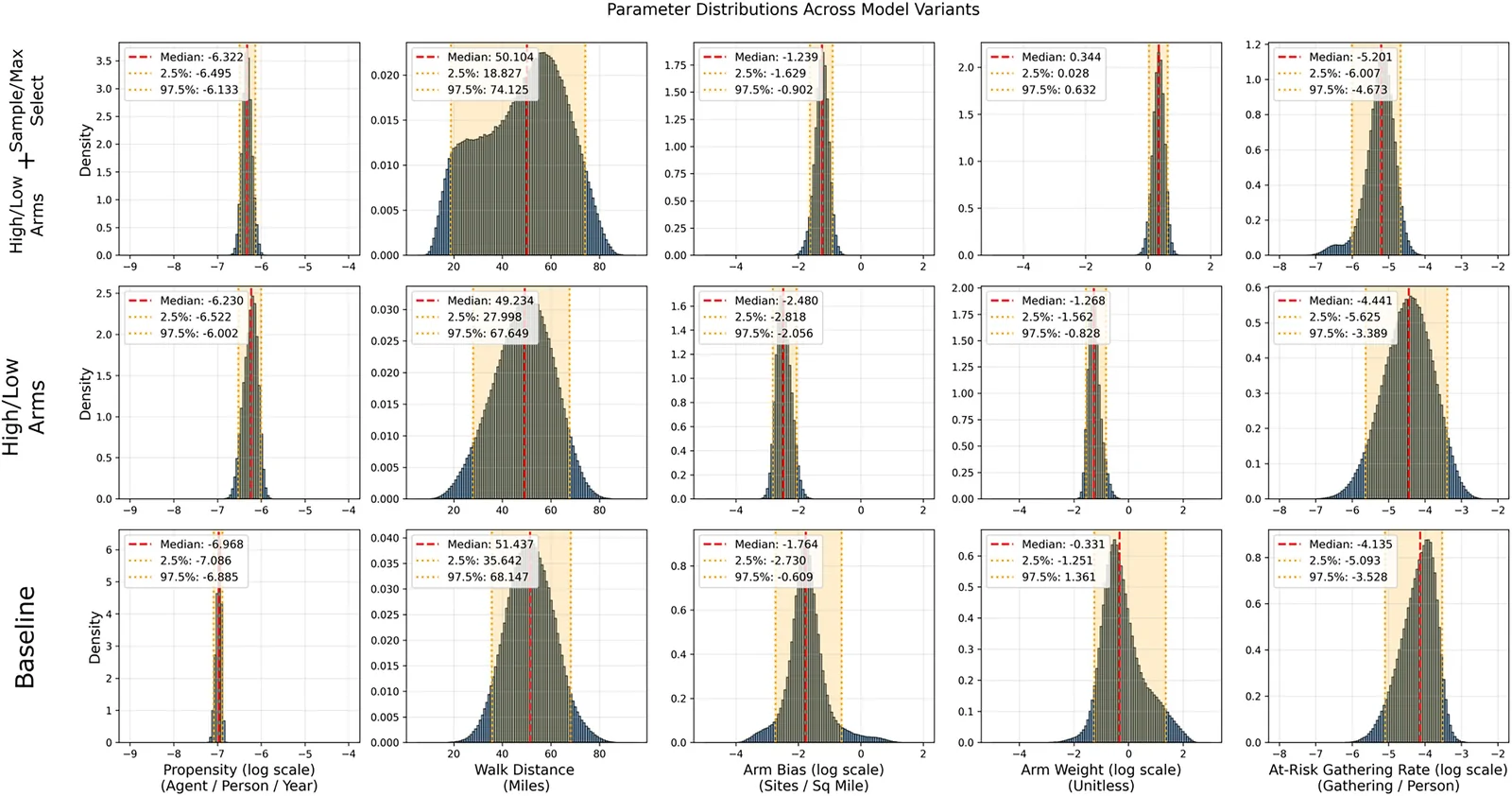
Posterior distribution of ABM parameters approximated by VBMC. Grid of 15 histograms (3 model variants by 5 parameters) showing VBMC posterior distributions, each marked with its median and 2.5%/97.5% quantiles. Rows: Baseline, High/Low Arms, High/Low Arms + Sample/Max Select. Columns: Propensity, Walk Distance, Arm Bias, Arm Weight, At-Risk Gathering Rate. Walk distance displayed in real units, while all other parameters are displayed in log-space. Distribution estimates shown for each of Baseline, High/Low weapon modeling, and High/Low weapon modeling + Max Select agent mixture.

VBMC provides us with an approximate posterior distribution for critical ABM parameters across the three model variants (Fig. 2). Because our ABM model is grounded in real-world data, parameters approximately correspond to real-world variables. However, they may still differ significantly depending on modeling assumptions.

Baseline agent propensity is robustly predicted to be between 1e−6 and 1e−7 agents/person/year, indicating an extremely low percentage of motivated agents each of our modeled classes. Other parameters are less tightly determined, with particular differences in magnitude for the at-risk gathering rate and walk distance parameters depending on choice of framework.

**Figure 3:**
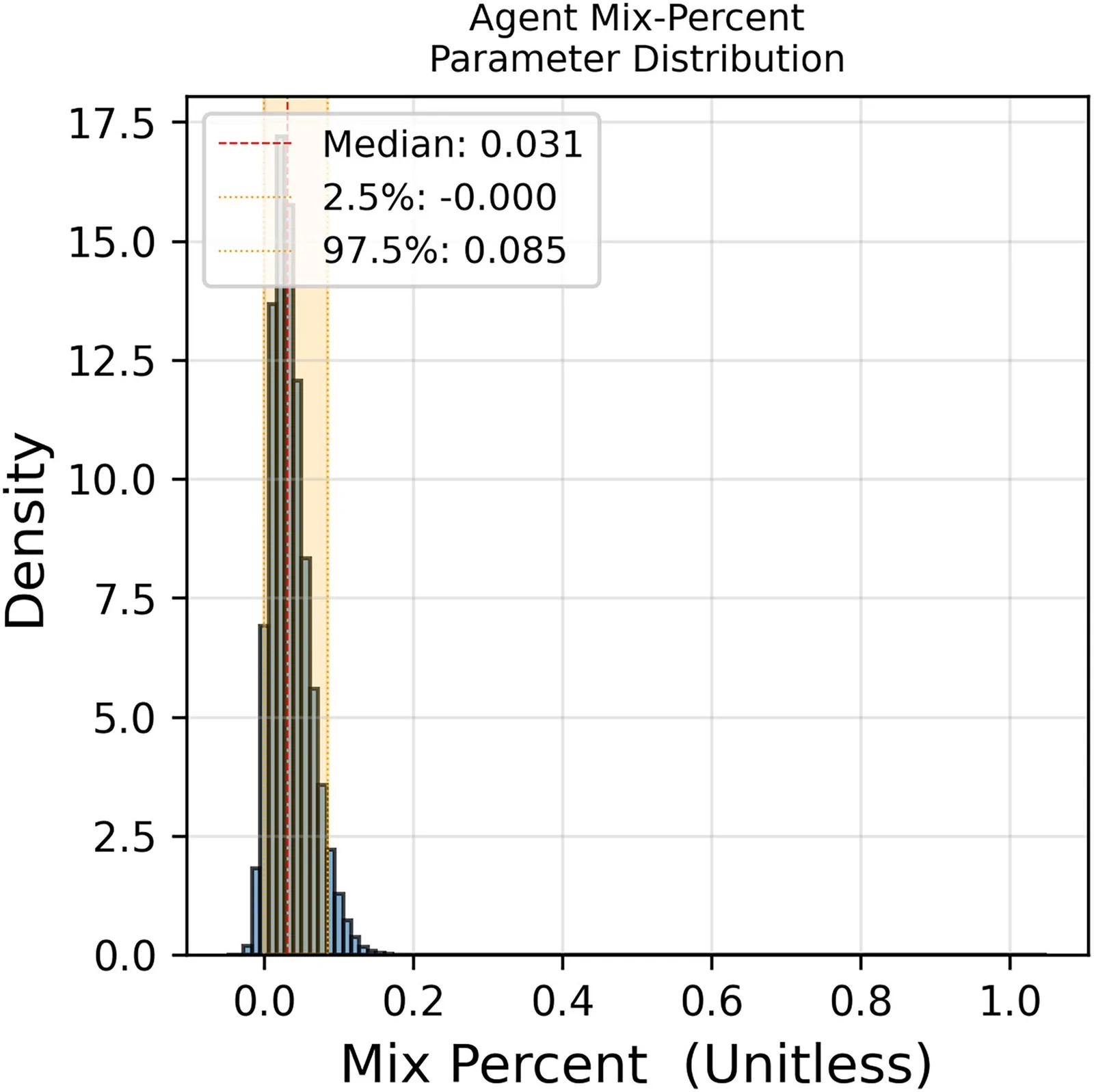
Posterior distribution of max-select probability parameter of High/Low firearm + Max Select simulations. Max-select probability is concentrated at low levels (Mean 0.031), indicating high rarity of max-select behavior under simulation assumptions.

Our model including the chance of a max-select agent indicates an extremely low likelihood of such behavior (Fig. 3), supporting prior research which indicates that most mass shooters select targets they are already familiar with (21). However, manual review of the most significant incidents on record suggest rare outlier behavior (4, 6), indicating that a complete treatment of mass shooting events will likely require a more finely tuned model of potential agent behavior, or custom handling of such scenarios in some capacity.

### WAIC eval

The four models defined above are compared using the Widely Applicable Information Criterion (WAIC), reported in Table 1.

**Table 1.** Model comparison using WAIC. Higher (less negative)WAIC indicates better model fit, with standard error indicating spread of potential WAIC evaluations under bootstrapping.

Model	LPPD	WAIC corr.	WAIC	SE
High/Low Arm + Max Sel.	− 1,143.47	3.25	− 1,146.71	0.2760
High/Low Arm	− 1,140.97	3.08	− 1,144.05	0.2753
Baseline ABM	− 1,173.36	5.12	− 1,178.48	0.2819
Baseline Naive	− 1,199.40	2.51	− 1,201.91	0.2854

We find that accounting for high-severity weapons significantly increases the log pointwise predictive density (LPPD) and WAIC score. However, we find that modeling max-select shooters slightly decreases overall performance, indicating that this behavior is sufficiently rare, and distorts statistics so strongly, that it typically does not explain real-world events. For subsequent evaluation, we use the High/Low firearm model, which achieves the highest overall performance on our dataset. Although it shows comparable performance to models accounting for max-select behavior, it outperforms them by several standard deviations on the WAIC metric, indicating a significantly better match to data.

All ABM models strongly outperform our baseline statistical model, indicating the successful integration of firearms and population data to explain mass shooting events. Each correctly learns parameters which differentiate locations with prior mass shooting events from baseline areas.

The relative ordering of these four models is robust to the structural choices that go into the WAIC computation. SI Appendix Section S3.2 reports WAIC for each ABM family under one-at-a-time perturbations of the severity scale, the Weibull gathering scale and shape, and (where defined) the per-encounter high-weapon probability. Across every perturbation tested, the High/Low Arm and High/Low Arm + Max-Select models outperform the Baseline ABM by 25–40 WAIC units, and the Max-Select extension remains 2–5 WAIC units below the High/Low Arm reference. No perturbation reorders the families, so the model-comparison conclusions reported above are not artifacts of any single structural choice.

### Posterior parameter distributions

**Figure 4:**
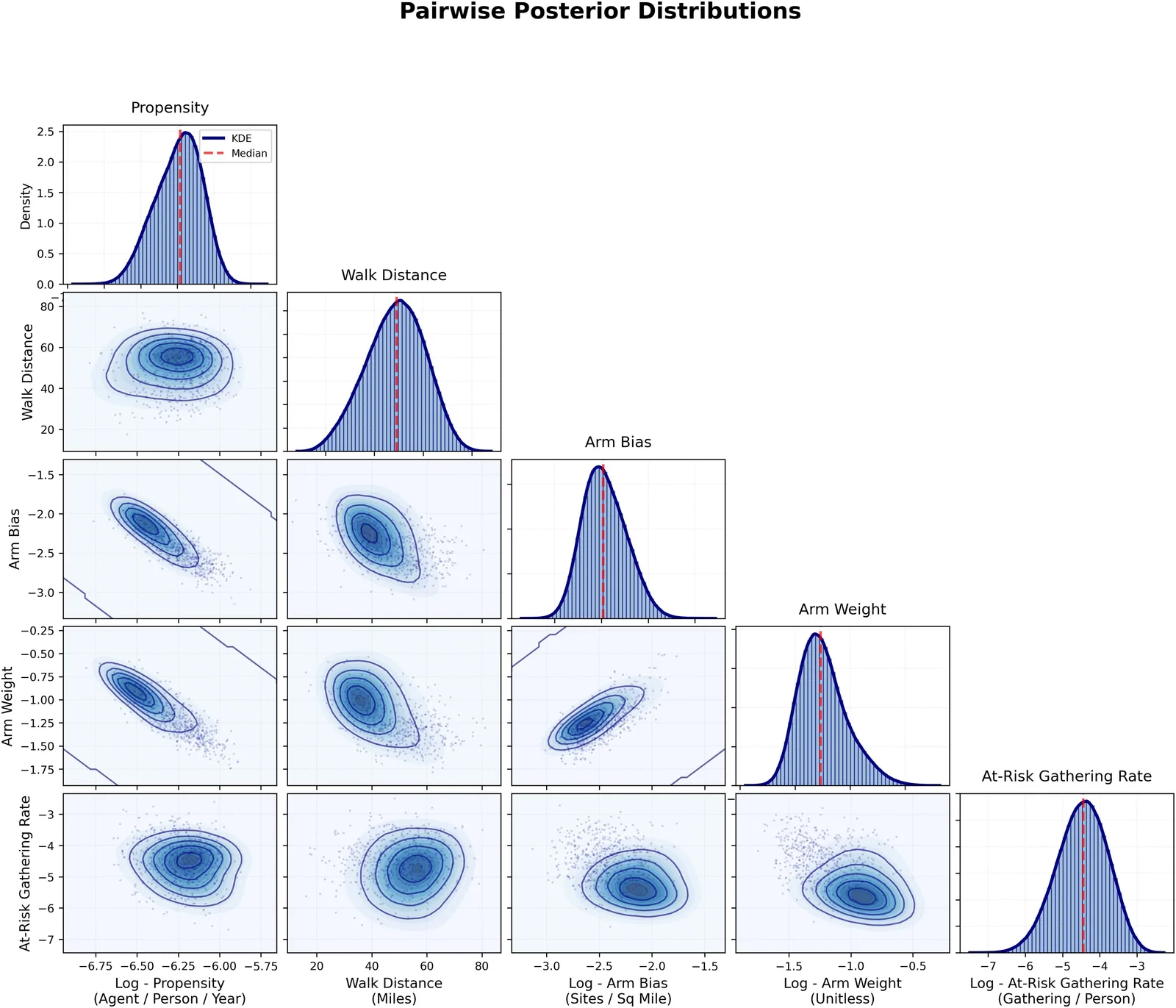
Corner plot of pairwise posterior distributions for five ABM parameters: log Propensity, Walk Distance (miles), log Arm Bias, log Arm Weight, and log At-Risk Gathering Rate. Diagonal panels show histograms with KDE curves and median lines; off-diagonal panels show 2D KDE contours over scatter samples. Propensity is negatively correlated with Arm Bias and Arm Weight, Arm Bias and Arm Weight are positively correlated, and Walk Distance and Gathering Rate are roughly uncorrelated with the others. Walk distance displayed in real units, while all other parameters are displayed in log-space. Marginal distributions estimated through 1e6 samples from VBMC mixture model fit to dataset log-likelihood.

The posterior distribution over parameters indicates that propensity and walk distance have a relatively strong negative correlation between firearm bias and firearm density, with at-risk gathering rate showing a similar, but much weaker trend (Fig. 4). Firearm bias and firearm weight exhibit a positive marginal posterior correlation, however, this dependence vanishes after conditioning on the remaining parameters, indicating that this is due to their shared role in the calculation of firearm density within the simulation process. Relatively low correlation between walk distance and at-risk gathering rate indicates a limited marginal effect of gathering rates in the region of highest probability.

### Posterior risk profiles

Conditional on sampled posterior parameters, our ABM simulations produce both the frequency and severity distributions of events meeting the mass-shooting criteria across US regions. For each subcounty, we quantify risk in two ways: (i) the expected probability of at least one mass shooting per decade and (ii) the expected number of fatalities per capita per decade.

These risk distributions are broadly similar and are both notably higher in areas with historically observed shootings. This indicates that the learned posterior distribution over ABM parameters successfully differentiates high- and low-risk areas even after controlling for population size. Across all subcounties, predicted risk ranges from ≈0.1 to 4 fatalities per million residents per decade, implying wide regional variation.

**Figure 5:**
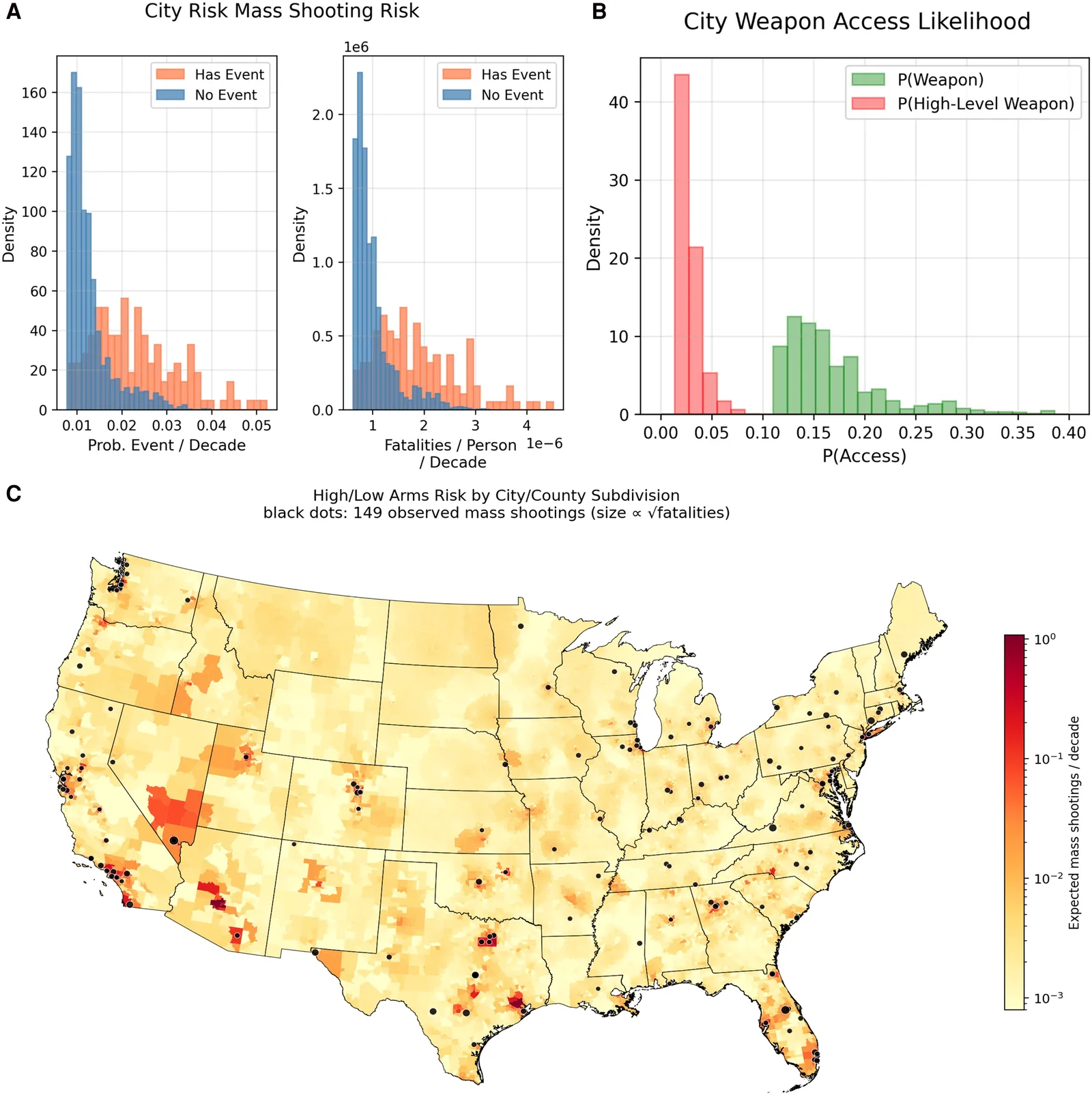
A) Histograms of predicted probability of mass shooting event per decade, and expected fatalities per person per decade across American regions. B) Histogram of predicted likelihood of agent acquiring firearms, or acquiring high-level firearms, across American regions. C) Heatmap of predicted probability of mass shooting event per decade across American subcounty regions, plotted against a scatterplot of observed events.

A significant limiting factor on risk rate for all simulations is acquisition of a firearm. Analysis of firearm acquisition probabilities reveals that firearm access remains the limiting factor in simulated event rates. Across regions, only 10–40% of motivated agents successfully acquire any weapon, and fewer than 10% obtain high-lethality firearms (Fig. 5B). These results suggest that even considering the widespread availability of firearms in America, acquiring a weapon is still a strong limiting factor on mass shooting events.

We take the additional step of predicting mass shooting risk across counties in America. We find that predicted risk varies significantly across regions, and qualitatively matches well with the geographic locations of observed events (Fig. 5C). The 10 highest-risk subdivisions by expected events per decade and by per-capita fatalities per decade are reported in Tables S5 and S6, alongside quantile summaries of predicted risk across all 5,057 scored subdivisions (Table S4).

**Figure 6:**
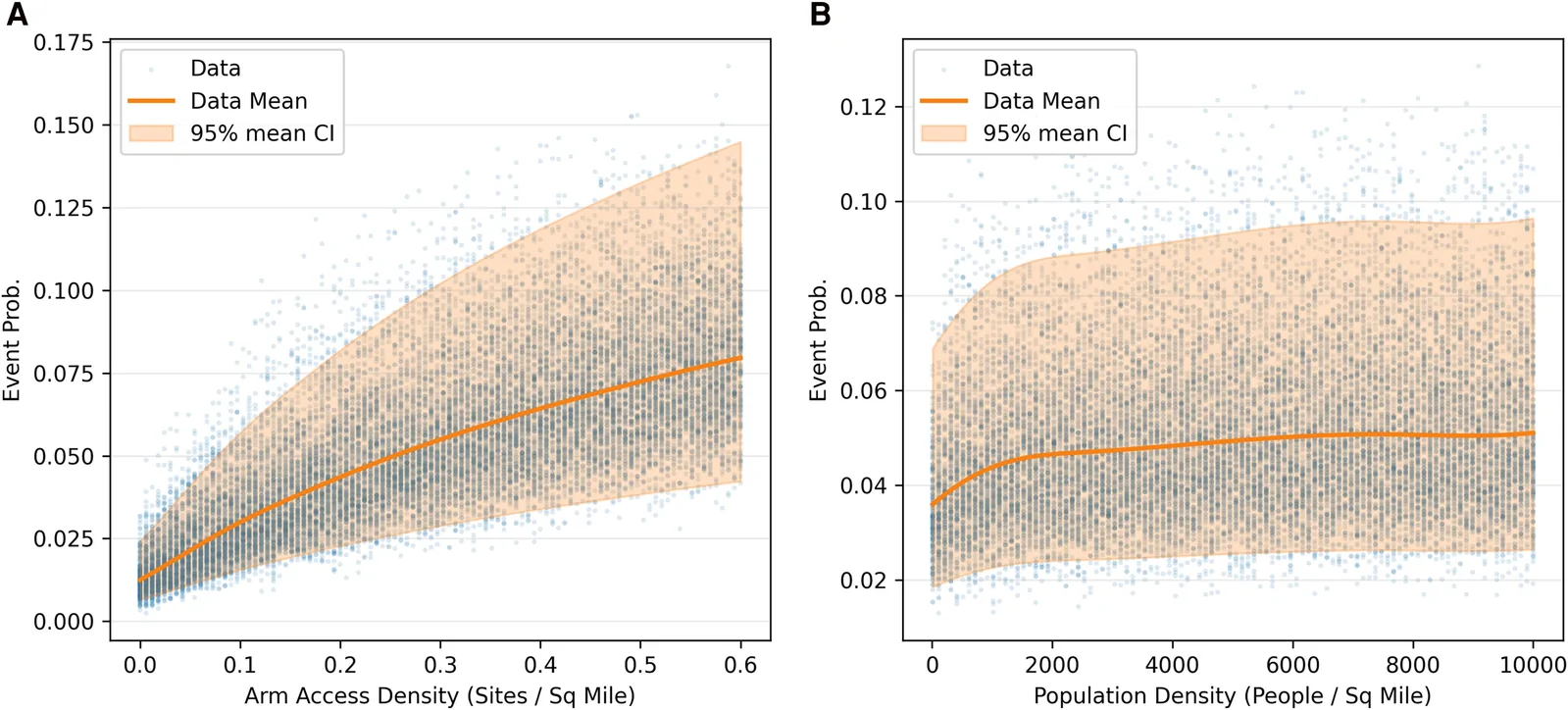
Mass shooting probability per decade, as estimated by the posterior distribution over ABM models, plotted against A) FFL density and B) population density. 90% model CI shaded.

In Fig. 6, we take the additional step of quantifying the causal effect of FFL density and population density on mass shooting risk within the High/Low firearm access ABM. While mass shooting risk is simply assumed to be directly proportional to population size, the effect of FFL density and population density is less straightforward to evaluate. We evaluate the predicted causal effect of each of these factors by repeatedly sampling parameters from our model posterior and calculating the per decade probability of an event in a representative city with varying values of either variable. We find that FFL density is predicted to have the most significant effect, with a very high marginal rate of increase for mass shooting risk as it is increased from zero. Population density is found to have a limited effect on risk, with its effect saturating quickly at higher density levels in our framework.

## Discussion

The first contribution of our work is a quantitative predictive claim, that the calibrated High/Low Arm ABM fits the observed subcounty event distribution at least as well as comparable statistical baselines on the same demographic inputs, achieving ΔWAIC=+57.86 against the Poisson–Weibull naive baseline (Table 1; −1144.05 vs. −1201.91, both with bootstrap SE ≈0.28). The second is a mechanistic claim: that the internal dynamics of the ABM are grounded enough across plausible structural specifications to support counterfactual reasoning about shooter behavior, and unobservable environmental factors. This is the stronger claim; we support it through the systematic sensitivity sweep reported in SI Appendix Section S3, which refits the reference model end-to-end under one-at-a-time perturbations of the per-encounter high-weapon probability, the simulation grid resolution, the FFL access-radius and firearm-availability proxy, the severity scale, and the Weibull gathering-size shape and scale. Across all perturbations, relative model performance is preserved, and most parameters are robustly estimated within the same order of magnitude, indicating that our conclusions are not artifacts of any single structural choice.

The posterior over our ABM reproduces observed patterns in the frequency and severity of US mass shootings while maintaining interpretability and statistical grounding. Despite the technical difficulty of applying Bayesian inference to agent-based simulations of rare events, our framework demonstrates that such calibration is feasible when supported by high-throughput likelihood evaluation. The result is a tractable, interpretable approach for linking mechanistic simulation with probabilistic modeling, which can be directly compared to existing statistical models.

Empirically, firearm access, proxied by Federal Firearms License (FFL) density, emerges as a dominant predictor of mass shooting risk, while population density shows only weak, saturating effects. In all of our ABM specifications, the inferred per-capita propensity for perpetrator emergence is extremely low ($10^{-6}$–$10^{-7}$ per person per year), consistent with the rarity of such actors. This estimate provides a quantitative basis for the understanding that while grievances or mental health crises may be relatively common, the transition to a “motivated perpetrator” of targeted mass violence is an exceptionally rare outcome (21, 22).

These parameters ground prior qualitative findings in quantitative, generative terms, showing that both firearm access and motivational factors are necessary conditions for events to arise. This finding matches a substantial body of empirical research linking measures of firearm availability, such as higher gun ownership rates or the presence of permissive state laws, to increased rates of firearm violence, including mass shootings (1, 14, 31). Our work provides a mechanistic complement to these statistical associations by demonstrating a plausible generative process where local access (proxied by FFLs) directly modulates the probability of a motivated agent successfully acquiring a weapon.

Because model parameters correspond to interpretable societal quantities, they can be modified to test hypothetical interventions. In all tested models, lowering firearm access or reducing the effective radius of weapon acquisition substantially decreases simulated event probabilities. As in other areas of public health and criminology, in silico experimentation opens the possibility of applying ABMs to evaluate policy outcomes where real-world experiments are infeasible or unethical (10, 11). For example, future models may directly integrate specific effects of policies like universal background checks or “red flag” laws by mechanistically altering firearm access or propensity parameters, providing policymakers with a range of probable outcomes.

Several limitations remain. Simplified behavioral assumptions, such as homogeneous agent behavior, or constant parameters across time or geography, constrain realism. In order to keep model fitting tractable, given the extremely limited dataset, we make several strong assumptions about the space of plausible models, including sampling the size of at-risk populations from a fixed Weibull distribution, assuming a fixed baseline rate for at-risk populations, and assuming random walk behavior from simulation agents.

There is strong evidence that mass killings can cluster in time, suggesting a copycat effect that our current model does not capture (36). We deliberately omit explicit contagion dynamics because coupling events across time breaks the per-event Poisson assumption that makes our likelihood-based calibration tractable; prior statistical work in this area uses the same simplification (4, 30). Extending the present framework to capture temporal clustering is a natural direction for future work. In the underlying framework of dataset likelihood evaluation, dozens of different models could be applicable and could strengthen the predictive power of results.

Moreover, reliance on contemporary FFL distributions as a proxy for historical access introduces temporal bias. We also investigate the use of longitudinal data on other gun-access proxy variables, notably the RAND household firearm survey and the firearm-share-of-suicides (FS/S) ratio derived from CDC WONDER and the NCIPC injury dashboard (37–39) and find that the FFL-based model achieves a substantially better fit to data. In practice, we find that high spatial resolution FFL data substantially improves model fit. We rerun the full calibration with both the RAND household-firearm rate as a time-varying alternative proxy, and report results alongside the FFL-based calibration in SI Appendix Section S3 (Tables S1–S3). The FFL-based model achieves a substantially better fit to the data (*Δ*WAIC of ≈−9.7 for the RAND-proxy refit relative to the FFL baseline). We additionally rerun every ABM family using a county-level FS/S proxy assembled from CDC WONDER and the NCIPC dashboard (SI Appendix Section S5, Table S7); the FFL calibration outperforms FS/S by 19–28 ELPD units for every family, and, critically, the relative ranking of ABM families is preserved under both proxies (High/Low Arm > Max-Select > Baseline). Switching to the longitudinal ratio does not change which model is preferred, only the absolute fit quality. Unfortunately, FFL data are not available across the full historical window, with no single proxy winning the spatial-resolution/temporal-coverage trade-off cleanly.

Within the FFL-radius sensitivity sweep (SI Appendix Section S3), tighter access buffers fit the data progressively better, with the 7.5 mi configuration achieving the best fit across all conditions tested. We interpret this as evidence that local firearm-marketplace density carries more predictive signal than wider regional aggregates, consistent with a generative process in which a perpetrator’s purchase opportunities are dominated by their immediate neighborhood rather than a county- or regional-scale firearm climate.

Despite these constraints and limitations, log-likelihood and WAIC comparisons show that applying Bayesian inference to ABMs can indeed produce models that meaningfully predict real-world data. The approach offers a framework for the principled integration of real-world data into mechanistic models, and a consistent methodology for choices between competing models.

## Conclusion

This study demonstrates the potential of Bayesian parameter estimation to calibrate mechanistic, ABMs, to rare, real-world events. Importantly, this framework is flexible and extensible, allowing for future improvements as new data sources and modeling approaches emerge. Bayesian learning, and evaluation, of ABM models for mass shootings enables the empirical evaluation of high-level models for the causes and risk-factors for mass shootings, and the calibrated estimation of parameters with real-world impact, such as the marginal impact of firearms access, or the baseline propensity of actors toward potential violence. In doing so, this work establishes a quantitative bridge between simulation and data, advancing the prospect of mechanistic social models that can be statistically tested, compared, and ultimately used to inform evidence-based policy.

## Supplementary Material

pgag294_Supplementary_Data

## Data Availability

All data used in this study is publicly available through the Mother Jones mass shooting database (5, 27), the Federal Firearms Listings database (29), and NHGIS (23). All code for analysis is publicly available at github.com/mofradlab/gv_abm.
