# Supplementary material for: Forecasting the future of American mass shootings with Bayesian agent-based model calibration
Source: PNAS Nexus. 2026 Sep 22;5(9):pgag294. doi: 10.1093/pnasnexus/pgag294 (PMC13595443; doi:10.1093/pnasnexus/pgag294)
Supplement: Supplementary file 1 [file pgag294_supplementary_data.pdf]

Forecasting the future of American mass shootings with Bayesian agent-based model calibration — Supplementary Information

Andrew M. Dickson[1], Mira Bhatt[1], Tiffany Yu[1], Mohammad R.K. Mofrad[1,2,*]

[1]Departments of Bioengineering and Mechanical Engineering, University of California, Berkeley, CA 94720, USA

[2]Molecular Biophysics and Integrative Bioimaging Division, Lawrence Berkeley National Lab, Berkeley, CA 94720, USA

*Corresponding author: mofrad@berkeley.edu

August 21, 2026

SI Appendix

S2.1 Grenander Estimator for Severity Distribution

We assume that the probability density function (PDF) of mass shooting severity, conditional on the existence of a motivated perpetrator, is monotonically decreasing. This is true by construction for each considered ABM framework. Let S denote the number of fatalities resulting from a simulated event and let $F_N(s)$ represent the empirical cumulative distribution function (CDF) obtained from N ABM simulations. The Grenander estimator [4] provides a nonparametric maximum-likelihood estimate of a decreasing density by taking the derivative of the smallest concave majorant of $F_N(s)$:

$$\hat{f}_N(s) = -\frac{d}{ds}\,\mathrm{SCM}(F_N(s))\,, \tag{1}$$

where $\mathrm{SCM}(\cdot)$ denotes the least concave majorant operator. This estimator guarantees a valid monotone density and captures heavy-tailed behavior in the severity distribution. Empirically, we find that $N \approx 5 \times 10^4$ conditional ABM samples per sub-county yield stable estimates of the upper tail, including events exceeding 100 fatalities. The resulting estimator is normalized to ensure that $\int_0^\infty \hat{f}_N(s)\,ds = 1$.

The probability of an event exceeding the mass shooting threshold (≥ 3 fatalities) is then:

$$p_{c_i \geq 3} = \int_3^\infty \hat{f}_N(s \mid \theta_{\mathrm{city}_i}, \theta_{\mathrm{general}})\,ds, \tag{2}$$

which enters the likelihood as the event detection probability for each simulated location.

S2.2 Full Likelihood Derivation

Let $\mathcal{D}$ represent the dataset of all sub-counties c_i, each associated with a population $c_{i,\mathrm{pop}}$, and a set of recorded event severities $\vec{E}_i = \{x_{i1}, \ldots, x_{iN_i}\}$. Conditioned on the global parameters $\theta_{\mathrm{general}}$ and sub-county–specific parameters θ_{city_i}, the generative model defines the expected number of motivated agents as

$$\mu_i = \lambda_{\mathrm{prop}}\, c_{i,\mathrm{pop}}\, T\, p_{c_i \geq 3},$$

where $p_{c_i \geq 3}$ is given by Eq. 2. The probability of observing the complete dataset $\mathcal{D}$ is then

$$P(\mathcal{D} \mid \theta_{\mathrm{general}}) = \prod_{i \in \mathcal{D}} \left[\frac{\mu_i^{N_i} e^{-\mu_i}}{N_i!} \prod_{j=1}^{N_i} \frac{1}{p_{c_i \geq 3}} f_{\theta_{\mathrm{city}_i}}(x_{ij} \mid \theta_{\mathrm{general}}) \right], \tag{3}$$

where the first term represents the Poisson likelihood of event counts, and the second term represents the conditional likelihood of observed severities given that an event occurred. The normalization by $p_{c_i \geq 3}$ ensures that only events meeting the reporting threshold contribute to the probability mass.

The posterior distribution over parameters is then obtained by Bayes' rule:

$$P(\theta_{\text{general}} \mid \mathcal{D}) \propto P(\mathcal{D} \mid \theta_{\text{general}})\, P(\theta_{\text{general}}), \tag{4}$$

where the prior term $P(\theta_{\text{general}})$ is defined as below. Posterior inference is performed using Variational Bayesian Monte Carlo (VBMC), which approximates the posterior density as a Gaussian mixture model by iteratively querying the log-likelihood surface. Convergence is tested using a combination of heuristics from the PyVBMC package, specified in detail in the original implementation [1, 2].

S2.3 Prior Specification

Parameters are normalized to the [0, 1] range, and then linearly transformed following sampling. The walk distance and, for max select simulations, mix_rate are input directly to models, while all other parameters are input following an exponential transform. Parameters are sampled from a large range, but bounded by strong priors, with Beta(5, 5) priors applied to the normalized range.

Posterior summaries reported in the main text are derived from VBMC sampled until convergence of the surrogate model (˜1,200 samples in practice).

WAIC scores are computed from 500 Monte Carlo evaluations of the log-likelihood per model to ensure numerical stability under data weighting.

S2.4 Downsampling

Sampling probabilities for datapoints were a function of population density, population, and FFL density, and assigned inverse probabilities as sample weights.

$$w_i = \frac{1}{p_i}. \tag{5}$$

We computed a softmax-based weighting function for population density, gun density (approximated by Federal Firearms License (FFL) counts per capita), and County population. While the sampling functions differed in formula, they followed a basic equation of

$$w_i = 2 \cdot \left(\text{softmax}\left(\frac{x_i}{s}\right) - 0.5\right), \tag{6}$$

where s is a sensitivity scaling constant. We then took the average of each variable specific probability

$$p_i = \frac{1}{3}\left(p_i^{(\text{pop})} + p_i^{(\text{popdens})} + p_i^{(\text{gun})}\right), \tag{7}$$

and set $p_i = 1$ for locations that experienced a mass shooting.

These transformations ensured that sub-counties with larger populations or population densities, or higher firearm availability received higher sampling probabilities, without biasing log-likelihood evaluations.

Weights ensured that the downsampled dataset matched the expected values of the complete dataset in population, population density, and firearm density.

0.1 S2.5 ABM Local Population Density

Average population for ABM simulations is known from sub-county level data, but local population at specific grid cells is modeled as a sum of a Poisson distributed number of two-dimensional Gaussians, with a mean of five per simulation instantiation. Gaussian means are sampled from a uniform distribution over simulation cell centers, and Gaussians are assumed to have standard deviation of 2.5 miles in all directions.

Grid cell population density is assigned based on the total density of the Gaussian mixture at their geographic center, with all densities normalized to match the total population density of the area.

0.2 S2.6 Programming Environment

All code written in Python 3.10, using the Pandas [15], NumPy [11], Jax [5], PyMC [1], statsmodels [18], and PyVBMC [12] packages.

0.3 S2.7 Dataset Availability

All data used in this study is publicly available through Mother Jones [14, 10], the Federal Firearms Listings database [6], and NHGIS respectively [17]. All code for analysis is publicly available at github.com/mofradlab/gv_abm.

S3 Structural Sensitivity Analyses

We refit the High/Low Arm reference model under one-at-a-time perturbations of five structural choices: the per-encounter high-weapon access probability, the simulation grid resolution, the FFL access-radius and the firearm-availability proxy, the severity-scale parameter, and the Weibull gathering-size shape and scale. Each refit re-runs the full VBMC calibration with the perturbed setting fixed; baseline values are $p_{\text{hw}} = 0.10$, grid = 30, FFL radius = 15 mi, severity scale = 1.0, Weibull shape = 2.0, Weibull scale = 0.4. The narrow-scale Weibull condition (wsc = 0.30) is included as an out-of-range misspecification probe rather than as an empirically plausible alternative; lower scales compress simulated gathering sizes below empirically observed regimes (cf. Methods).

S3.1 Sensitivity WAIC summary

Table S1 reports the Widely Applicable Information Criterion (WAIC) computed for each sensitivity refit of the High/Low Arm reference model. WAIC values are computed using the same procedure as the main-text Table 1, with 500 Monte Carlo evaluations of the per-sub-county weighted log-likelihood, with the standard variance correction so that ΔWAIC values reported here are directly comparable to the High/Low Arm vs. Baseline Naive gap in the main text. Higher (less negative) values indicate better fit; ΔWAIC is signed so that positive values denote improvement over baseline.

The WAIC ranking reproduces the qualitative pattern observed in the converged VBMC ELBOs: the 7.5 mi FFL radius and the 0.5× severity-scale variants outperform baseline by ~ 9 WAIC units, while the RAND household-firearm proxy and the out-of-range narrow-Weibull probe (wsc = 0.30) sit ~ 10 units below. Within the empirically supported envelope of structural choices, every condition falls within roughly 2 WAIC units of baseline, comparable to or smaller than the WAIC standard error (~ 0.28). This supplies the formal predictive-fit support for the mechanistic-tier robustness claim in the Discussion.

S3.2 Cross-model WAIC under structural perturbations

Table S2 extends the sensitivity sweep of Section S3.1 to the two alternative ABM families compared in the main-text Table 1: the High/Low Arm + Max-Select extension and the Baseline ABM without high-weapon stratification. For each of the three model families, we recompute WAIC under perturbations of the severity scale (F), the Weibull gathering scale (G-scale), and — where applicable — the per-encounter high-weapon probability. Each ΔWAIC is computed within-model against that family's own baseline value, so that within-row comparisons isolate the effect of the perturbation, while column-wise comparisons isolate the model-family effect. WAIC standard errors are omitted from non-baseline rows because they vary by less than 0.02 across the sweep.

The ranking of model families is preserved across nearly every perturbation: the High/Low Arm reference and its Max-Select extension outperform the Baseline ABM by 25–40 WAIC units in every cell, well above the SE-defined noise floor of ~ 0.28. The Max-Select extension remains 2–5 WAIC units below the reference under every condition tested, reproducing the main-text finding that max-select behavior is sufficiently rare to be neutral or mildly disfavored regardless of structural perturbation.

We find that the Baseline ABM is sensitive to the severity-scale (F) and Weibull-scale (G) parameters: the 0.5× severity perturbation closes 12.21 WAIC units of the gap to the reference, and the 0.50 Weibull-scale perturbation closes a striking 28.82 units. Even after these improvements, however, the Baseline ABM remains 5–7 WAIC units below the High/Low Arm reference, preserving the main-text

Task	Condition	WAIC ± SE	ΔWAIC
Baseline	High/Low Arm	−1144.05 ± 0.28	+0.00
Weapon Access	High-weapon prob = 0.05	−1144.19 ± 0.27	−0.14
Weapon Access	High-weapon prob = 0.20	−1145.93 ± 0.28	−1.88
Grid Resolution	Grid = 20 × 20	−1143.77 ± 0.28	+0.28
Grid Resolution	Grid = 40 × 40	−1143.12 ± 0.27	+0.93
FFL Access	FFL radius = 7.5 mi	−1134.56 ± 0.27	**+9.49**
FFL Access	FFL radius = 30 mi	−1145.93 ± 0.28	−1.88
FFL Access	RAND household-firearm proxy	−1153.71 ± 0.27	**−9.66**
Severity Scale	Severity scale ×0.50	−1134.78 ± 0.27	**+9.27**
Severity Scale	Severity scale ×1.50	−1150.14 ± 0.27	−6.09
Weibull Scale	Weibull scale = 0.30	−1154.45 ± 0.28	**−10.40**
Weibull Scale	Weibull scale = 0.50	−1143.46 ± 0.28	+0.59

Table S1: WAIC across the sensitivity sweep. Standard errors are bootstrap estimates from the WAIC variance-correction term; lower (more negative) WAIC is worse fit, so $\Delta\text{WAIC} > 0$ indicates improvement over baseline. Bold entries indicate the four conditions where $|\Delta\text{WAIC}| > 9$, well above the WAIC standard error.

ordering. We interpret this as evidence that a substantial fraction of the Baseline ABM's deficit at its registered baseline values is absorbed by the high-weapon stratum in the High/Low Arm framework, rather than reflecting a fundamental advantage of the Baseline ABM at any parameterization.

Third, the 0.30 narrow-Weibull shape degrades fit across all three models (−10.40, −13.93, −27.80), confirming that this perturbation is a genuine misspecification. The degradation scales roughly with each model's baseline severity-distribution flexibility, with the Baseline ABM suffering most.

Together, these results extend the mechanistic-tier robustness claim of Section S3.1 from the High/Low Arm reference to the broader model class: both family ranking and within-family parameter inference are stable across the structural perturbations tested.

Task	Sweep value	High/Low Arm	+ Max Sel.	Baseline ABM
Severity scale	baseline (×1.0)	−1144.05 ± 0.28	−1146.71 ± 0.28	−1178.48 ± 0.28
	×0.50	−1134.78 (+**9.27**)	−1144.25 (+2.47)	−1166.27 (+**12.21**)
	×1.50	−1150.14 (−6.09)	−1150.92 (−4.21)	−1170.69 (+7.78)
Weibull scale	baseline (0.40)	−1144.05 ± 0.28	−1146.71 ± 0.28	−1178.48 ± 0.28
	0.30	−1154.45 (−**10.40**)	−1160.64 (−**13.93**)	−1206.28 (−**27.80**)
	0.50	−1143.46 (+0.59)	−1151.52 (−4.81)	−1149.65 (+**28.82**)
High-weapon probability	baseline (0.10)	−1144.05 ± 0.28	N/A	N/A
	0.05	−1144.19 (−0.14)	N/A	N/A
	0.20	−1145.93 (−1.88)	N/A	N/A

Table S2: Cross-model WAIC under the structural perturbations of Section S3.1, evaluated for the three ABM families compared in the main-text Table 1. Cell entries are WAIC ± SE for baseline rows and WAIC followed by parenthetical ΔWAIC relative to that model's own baseline for perturbed rows; standard errors for perturbed rows fall in $[0.27, 0.29]$ throughout. Bold entries flag $|\Delta\text{WAIC}| > 9$, well above the WAIC SE. "N/A" marks perturbations that are not defined for the given model family (the Baseline ABM has no high-weapon stratum, and Max-Select inherits the high-weapon parameterization from the High/Low Arm reference).

S3.3 Posterior medians and 90% credible intervals per condition

Table S3 reports posterior medians and 90% credible intervals (5th, 95th percentile) for each of the five calibrated High/Low Arm parameters under every sensitivity refit. Conditions are grouped by sweep task to make within-task comparisons immediate. Read alongside Table S1: where parameters

shift substantially relative to baseline, the corresponding row in the WAIC table generally also fits substantially worse, so the conditions that significantly move the parameters are frequently the same conditions that fail to model the data competitively.

Within the empirically supported range of structural choices (excluding the strongly underperforming wsc = 0.30 probe and the alternative-proxy RAND row), all parameter medians shift by less than 0.5 in display units, supporting a robustness claim for the substantively interpreted parameters (per-capita propensity, FFL coefficients).

S4 City-Level Risk Predictions

Based on the posterior distribution over parameters obtained from the High/Low Arm reference model, we generate posterior-predictive risk estimates for every NHGIS county subdivision with population $\geq$ 10,000 in its latest available decade ($n = 5{,}057$ subdivisions). Each subdivision was scored against 100 posterior parameter draws from the High/Low Arm baseline, with 5,000 ABM simulations per draw, yielding posterior mean and standard deviation for events per decade, P(at least one event per decade), fatalities per decade, and fatalities per capita per decade. Of these 5,057 subdivisions, 67 have at least one mass shooting recorded in the Mother Jones dataset since 1982. These estimates represent the expected number of mass shooting events per decade, the probability of at least one event per decade, and the expected number of fatalities per decade and per capita per decade, under the fitted ABM dynamics.

S4.1 Distributional summaries

Table S4 reports quantiles of predicted events per decade and predicted fatalities per capita per decade across the 5,057 scored subdivisions. The long upper tail of events per decade reflects the concentration of risk in a small number of large, high-FFL-density subdivisions; the corresponding per-capita rate has a much narrower spread, indicating that most variation in absolute event counts is driven by population scale.

S4.2 Highest-risk subdivisions by expected events per decade

Table S5 reports the ten subdivisions with the highest posterior-mean expected events per decade. "Observed events" is the number of distinct mass shootings assigned to that NHGIS subdivision in the Mother Jones dataset since 1982; populous counties may contribute multiple subdivisions to the table.

S4.3 Highest-risk subdivisions by expected fatalities per capita

Table S6 reports the ten subdivisions with the highest posterior-mean fatalities per capita per decade. The list is dominated by Texas and Arizona subdivisions, consistent with their FFL densities being substantially above the national mean.

S5 Longitudinal alternative proxy: firearm-share-of-suicides

As a complement to the contemporary FFL-density proxy used for the headline calibration and to the RAND household-firearm proxy reported in Section S3, we additionally refit each ABM family using the *firearm-share-of-suicides* (FS/S) statistic as a longitudinal, county-resolved proxy for firearm prevalence. FS/S has the advantage of decade-level temporal coverage spanning the full study window (1979–2024) and complete geographic coverage at the county level after 1999, complementing the spatial resolution of the FFL data with longitudinal depth.

S5.1 Data sources

We assemble county-level mortality counts from three CDC publications that together cover the full 1979–2024 study period:

- **Compressed Mortality File, 1979–1998** (CDC WONDER) [8]. Death-certificate counts coded with the International Classification of Diseases, ninth revision (ICD-9), grouped here by intent and mechanism. For 1979–1998 we use the national-level breakdown by age and cause-of-death; sub-state aggregation at that resolution was not available in the export used for this revision and is treated as a limitation (see Section 0.3).
- **Underlying Cause of Death, 1999–2016** (CDC WONDER) [7]. Death-certificate counts coded with ICD-10, broken down by U.S. county (5-digit FIPS code) and cause-of-death subgroup. Counts below the CDC reporting threshold are suppressed in compliance with NCHS disclosure rules.
- **Mapping Injury, Overdose, and Violence Dashboard, 2019–present** (CDC, National Center for Injury Prevention and Control) [9]. County-by-year crude and Bayesian-modeled death rates per 100,000 for six injury intents. The two relevant intents for this study are the union of all suicide mechanisms (ICD-10 codes X60–X84) and suicide-by-firearm (X72–X74).

All three sources are publicly available through CDC; we redistribute only the derived per-county-per-decade proxy values (not the raw mortality counts) to comply with NCHS suppression policies for cells below the reporting threshold.

S5.2 Definition of the firearm-share-of-suicides statistic

For a given geography g and time window t, the firearm-share-of-suicides (FS/S) is the fraction of all suicide deaths in (g, t) that were committed with a firearm:

$$\text{FS/S}(g,t) \;=\; \frac{N_{\text{firearm suicide}}(g,t)}{N_{\text{all suicide}}(g,t)} \;\in\; [0,1]. \tag{8}$$

The numerator and denominator are defined using ICD codes as follows:

Era	Numerator (firearm suicide)	Denominator (all suicide)
1979–1998 (ICD-9)	E955.0–E955.4	E950–E959
1999–present (ICD-10)	X72, X73, X74	X60–X84

For each county and decade we aggregate across years using a count-weighted mean, which reduces to summing the per-year numerator counts (or rates) and the per-year denominator counts (or rates) before forming the ratio.

S5.3 Interpretation as a firearm-ownership proxy

The FS/S statistic has been used as a proxy for the prevalence of household firearm ownership in social-science research since at least the early 1990s [3, 13, 19, 16]. The intuition is that the choice of method conditional on a suicide attempt depends on which lethal means are accessible in the home, so the firearm fraction of completed suicides reflects relative firearm availability without requiring survey self-report. Azrael et al. [3] validated this proxy against the General Social Survey at the state level and found a correlation of $r = 0.80$ with self-reported household-firearm rates; Siegel et al. [19] report similar agreement for state-year panels back to 1981; and Schell et al. [16] use FS/S as one of four indicators in their state-level latent-factor estimate of household firearm-ownership rates (the RAND TL-354 dataset that we use as a separate proxy in Section S3). The advantages of FS/S over survey-based measures are (i) complete geographic coverage, (ii) year-resolution longitudinal availability, and (iii) independence from non-response and social-desirability biases that affect direct survey questions about firearm ownership.

S5.4 Limitations

Three limitations of the FS/S proxy bear on its use in this study:

1. **Population denominator.** FS/S measures prevalence among households where a suicide occurred, not population-wide prevalence. The conversion factor between the two has been shown to be reasonably stable across U.S. regions [3], but it is not invariant across time, so cross-decade comparisons are interpreted as measuring relative rather than absolute change in availability.

2. **Small-count suppression.** CDC suppresses cells with fewer than 10 deaths in WONDER (Underlying Cause of Death) and reports an "unstable rate" sentinel for the NCIPC dashboard when modeled estimates fail to stabilise. Counties for which either the numerator or denominator is suppressed for a given decade are left as missing in the released proxy file and filled by nearest-neighbor spatial interpolation across the surrounding counties at use time.

S5.5 WAIC comparison: FFL vs. FS/S

Table S7 compares the held-out Watanabe-Akaike information criterion (WAIC, the WAIC-decomposable component summed across sub-counties) of each ABM family when calibrated against the contemporary FFL-density proxy versus the longitudinal FS/S proxy. WAIC is reported both for the full 1980–2024 record and decomposed by decade so that any longitudinal advantage of FS/S over a static FFL snapshot would be visible as a positive ΔWAIC in the early decades.

Across the full record, the FFL-based calibration outperforms the FS/S-based calibration by 19–28 WAIC units for every ABM family ($\Delta \text{WAIC}_{\text{FS/S}-\text{FFL}} = -26.93, -27.77, -18.83$ for the High/Low Arm reference, the Max-Select extension, and the Baseline ABM respectively; all well above the bootstrap SE of ~ 0.4). The decade-resolved breakdown shows that the FFL advantage is concentrated in the 2000s and 2010s, when FFL coverage is most reliable; the 1990s actually slightly favor FS/S for two of the three families, but the difference is small ($|\Delta \text{WAIC}| \leq 2.4$) and dwarfed by the later-decade advantage of FFL.

Critically, the relative ranking of ABM families is identical under both proxies: the High/Low Arm reference outperforms the Max-Select extension which outperforms the Baseline ABM, by margins of comparable magnitude (~ 2.7 and ~ 32 WAIC units under FFL; ~ 3.5 and ~ 23 WAIC units under FS/S). Switching from FFL to FS/S therefore changes neither the qualitative model comparison nor the conclusion that high-spatial-resolution firearm-access data dominates lower-spatial-resolution longitudinal data, replicating the finding from the RAND-proxy condition (Section S3.1) on a fully county-resolved alternative.

References

[1] Oriol Abril-Pla, Virgile Andreani, Colin Carroll, Larry Dong, Christopher J. Fonnesbeck, Maxim Kochurov, Ravin Kumar, Junpeng Lao, Christian C. Luhmann, Osvaldo A. Martin, Michael Osthege, Ricardo Vieira, Thomas Wiecki, and Robert Zinkov. PyMC: A modern and comprehensive probabilistic programming framework in Python. *PeerJ Computer Science*, 9(e1516), 2023.

[2] Luigi Acerbi. Variational Bayesian Monte Carlo, November 2018. arXiv:1810.05558 [stat].

[3] Deborah Azrael, Philip J. Cook, and Matthew Miller. State and local prevalence of firearms ownership: Measurement, structure, and trends. *Journal of Quantitative Criminology*, 20(1):43–62, 2004.

[4] Lucien Birge. The Grenader Estimator: A Nonasymptotic Approach. *The Annals of Statistics*, 17(4):1532 – 1549, 1989.

[5] James Bradbury, Roy Frostig, Peter Hawkins, Matthew James Johnson, Chris Leary, Dougal Maclaurin, George Necula, Adam Paszke, Jake VanderPlas, Skye Wanderman-Milne, and Qiao Zhang. JAX: composable transformations of Python+NumPy programs, 2018.

[6] Firearms Bureau of Alcohol, Tobacco and Explosives. Federal firearms listings database. Available at: `https://www.atf.gov/firearms/listing-federal-firearms-licensees`, 2025. Accessed 2025.

[7] Centers for Disease Control and Prevention, National Center for Health Statistics. Underlying cause of death, 1999–2016. CDC WONDER online database, released 2017. Available at: https://wonder.cdc.gov/ucd-icd10.html, 2017. Accessed 2025.

[8] Centers for Disease Control and Prevention, National Center for Health Statistics. Compressed mortality file, 1979–1998. CDC WONDER online database. Available at: https://wonder.cdc.gov/cmf-icd9.html, 2024. Accessed 2025.

[9] Centers for Disease Control and Prevention, National Center for Injury Prevention and Control. Mapping injury, overdose, and violence dashboard. Available at: https://www.cdc.gov/injury-violence-data/data-vis/index.html, 2025. Accessed 2025; archival export retained alongside publication code.

[10] Mark Follman, Gavin Aronsen, Deanna Pan, and Maggie Caldwell. Us mass shootings, 1982-2018: Data from mother jones' investigation. *Mother Jones*, 14, 2018.

[11] Charles R. Harris, K. Jarrod Millman, Stéfan J. van der Walt, Ralf Gommers, Pauli Virtanen, David Cournapeau, Eric Wieser, Julian Taylor, Sebastian Berg, Nathaniel J. Smith, Robert Kern, Matti Picus, Stephan Hoyer, Marten H. van Kerkwijk, Matthew Brett, Allan Haldane, Jaime Fernández del Río, Mark Wiebe, Pearu Peterson, Pierre Gérard-Marchant, Kevin Sheppard, Tyler Reddy, Warren Weckesser, Hameer Abbasi, Christoph Gohlke, and Travis E. Oliphant. Array programming with NumPy. *Nature*, 585(7825):357–362, September 2020.

[12] Bobby Huggins, Chengkun Li, Marlon Tobaben, Mikko J. Aarnos, and Luigi Acerbi. PyVBMC: Efficient Bayesian inference in Python. *Journal of Open Source Software*, 8(86):5428, June 2023. arXiv:2303.09519 [stat].

[13] Gary Kleck. Measures of gun ownership levels for macro-level crime and violence research. *Journal of Research in Crime and Delinquency*, 41(1):3–36, 2004.

[14] MotherJones. Us mass shootings, 1982–2025. Available at: https://www.motherjones.com/politics/2012/12/mass-shootings-mother-jones-full-data/, 2025. Accessed 2025.

[15] The pandas development team. pandas-dev/pandas: Pandas, February 2020.

[16] Terry L. Schell, Samuel Peterson, Brian G. Vegetabile, Adam Scherling, Rosanna Smart, and Andrew R. Morral. State-level estimates of household firearm ownership. Technical Report TL-354, RAND Corporation, Santa Monica, CA, 2020.

[17] Jonathan Schroeder, David Van Riper, Steven Manson, Katherine Knowles, Tracy Kugler, Finn Roberts, and Steven Ruggles. Ipums national historical geographic information system: Version 20.0, 2025. [dataset].

[18] Skipper Seabold and Josef Perktold. statsmodels: Econometric and statistical modeling with python. In *9th Python in Science Conference*, 2010.

[19] Michael Siegel, Craig S. Ross, and Charles King. The relationship between gun ownership and firearm homicide rates in the united states, 1981–2010. *American Journal of Public Health*, 104(11):2098–2105, 2014.

**Task**	**Condition**	**Propensity** ($\log_{10}$)	**Walk radius (mi)**	**Arm bias** ($\log_{10}$)	**Arm weight** ($\log_{10}$)	**Gathering rate** ($\log_{10}$)
Baseline	Baseline	−6.23 [−6.52, −6.00]	49.20 [27.83, 67.57]	−2.48 [−2.82, −2.05]	−1.27 [−1.56, −0.83]	−4.44 [−5.62, −3.39]
B: high-weapon	$p_{\mathrm{hw}} = 0.05$	−6.43 [−6.63, −6.23]	48.29 [31.29, 64.30]	−2.20 [−2.45, −1.94]	−1.02 [−1.30, −0.71]	−4.34 [−5.39, −3.41]
	$p_{\mathrm{hw}} = 0.20$	−6.19 [−6.50, −6.03]	44.20 [20.15, 63.99]	−2.52 [−2.84, −2.17]	−1.25 [−1.59, −0.82]	−4.30 [−5.60, −3.19]
C: grid	20×20	−6.35 [−6.57, −6.13]	52.71 [37.08, 67.98]	−2.39 [−2.57, −2.16]	−1.17 [−1.55, −0.83]	−4.33 [−5.36, −3.63]
	40×40	−6.32 [−6.55, −6.10]	55.84 [37.41, 72.60]	−2.45 [−2.83, −2.03]	−1.24 [−1.51, −0.90]	−4.11 [−5.09, −3.30]
D: FFL / proxy	$r = 7.5$ mi	−6.10 [−6.34, −5.91]	47.43 [34.13, 62.77]	−2.63 [−2.85, −2.30]	−1.53 [−1.75, −1.25]	−4.83 [−6.13, −3.33]
	$r = 15$ mi	−6.25 [−6.45, −6.06]	61.74 [48.61, 75.06]	−2.63 [−2.89, −2.33]	−1.38 [−1.66, −1.11]	−4.53 [−6.06, −3.27]
	$r = 30$ mi	−6.45 [−6.67, −6.15]	52.07 [32.96, 69.61]	−2.27 [−2.62, −1.91]	−0.99 [−1.47, −0.57]	−4.38 [−5.50, −3.42]
	RAND proxy	−6.48 [−6.84, −6.23]	46.07 [25.56, 67.77]	−1.88 [−2.42, −1.36]	−1.98 [−3.12, −1.49]	−4.05 [−4.82, −3.50]
F: severity	× 0.5	−6.39 [−6.56, −6.21]	51.68 [28.99, 71.90]	−2.16 [−2.51, −1.81]	−0.91 [−1.36, −0.48]	−4.47 [−5.60, −3.45]
	× 1.5	−6.36 [−6.60, −6.11]	53.66 [35.78, 69.88]	−2.41 [−2.70, −2.11]	−1.19 [−1.57, −0.80]	−4.54 [−5.83, −3.45]
G: Weibull shape	shape = 1.5	−6.33 [−6.62, −6.09]	48.30 [31.01, 63.31]	−2.28 [−2.64, −1.90]	−1.00 [−1.40, −0.57]	−4.48 [−6.17, −3.40]
	shape = 2.5	−6.20 [−6.51, −6.01]	54.39 [37.73, 69.94]	−2.65 [−2.93, −2.28]	−1.40 [−1.63, −1.02]	−4.42 [−5.80, −3.32]
G: Weibull scale	scale = 0.30	−6.15 [−6.43, −5.88]	46.62 [29.09, 65.16]	−2.68 [−3.01, −2.27]	−1.36 [−1.71, −1.00]	−4.46 [−5.80, −3.48]
	scale = 0.50	−6.62 [−6.78, −6.49]	47.87 [26.49, 68.68]	−1.99 [−2.44, −1.55]	−0.64 [−0.94, −0.22]	−3.96 [−5.15, −3.07]

Table S3: Posterior medians and 90% credible intervals (5th–95th percentile) for the five High/Low Arm parameters across all sensitivity-scan conditions. Log-scale parameters are reported in $\log_{10}$ display units; an interval of width 1 in $\log_{10}$ corresponds to a 10× change in natural units. Walk radius is in miles. Each row is summarised from 200,000 samples drawn from the corresponding VBMC variational posterior.

Percentile	events / decade	fatalities / capita / decade
50th	0.0021	9.6×10^{-7}
75th	0.0047	1.3×10^{-6}
90th	0.0112	1.5×10^{-6}
95th	0.0210	1.7×10^{-6}
99th	0.0805	2.0×10^{-6}

Table S4: Quantiles of posterior-predictive risk across the 5,057 scored subdivisions. Mean events per decade is 0.0069 and median 0.0021.

Rank	County, State	Population	Observed events	Expected events / decade	P(≥ 1 event / decad
1	Maricopa County, AZ	3,332,620	0	1.079 ± 0.197	0.6
2	Harris County, TX	3,465,454	0	0.983 ± 0.166	0.6
3	Dallas County, TX	1,608,717	0	0.414 ± 0.071	0.3
4	Los Angeles County, CA	2,561,253	0	0.367 ± 0.046	0.3
5	Kings County, NY	2,736,049	0	0.358 ± 0.053	0.3
6	Clark County, NV	2,005,713	2	0.326 ± 0.047	0.2
7	Orange County, CA	1,689,479	1	0.321 ± 0.045	0.2
8	Queens County, NY	2,405,834	0	0.306 ± 0.038	0.2
9	Cook County, IL	2,746,365	1	0.278 ± 0.050	0.2
10	Philadelphia County, PA	1,603,788	1	0.277 ± 0.037	0.2

Table S5: Top ten NHGIS subdivisions by posterior-mean expected mass-shooting events per decade. Posterior-mean ± std reported; $P(\geq 1 \text{ event / decade}) = 1 - \exp(-\lambda)$.

Rank	County, State	Population	Observed events	Fatalities / capita / decade
1	Maricopa County, AZ	3,332,620	0	$3.42 \times 10^{-6} \pm 6.2 \times 10^{-7}$
2	Denton County, TX	141,196	0	$3.16 \times 10^{-6} \pm 6.0 \times 10^{-7}$
3	Tarrant County, TX	615,586	0	$3.04 \times 10^{-6} \pm 5.8 \times 10^{-7}$
4	Harris County, TX	3,465,454	0	$3.01 \times 10^{-6} \pm 5.5 \times 10^{-7}$
5	Denton County, TX	111,754	0	$2.89 \times 10^{-6} \pm 5.2 \times 10^{-7}$
6	Tarrant County, TX	555,064	0	$2.87 \times 10^{-6} \pm 5.2 \times 10^{-7}$
7	Dallas County, TX	1,003,311	1	$2.83 \times 10^{-6} \pm 4.9 \times 10^{-7}$
8	Denton County, TX	273,304	0	$2.82 \times 10^{-6} \pm 5.2 \times 10^{-7}$
9	Maricopa County, AZ	509,957	0	$2.74 \times 10^{-6} \pm 4.9 \times 10^{-7}$
10	Dallas County, TX	1,608,717	0	$2.69 \times 10^{-6} \pm 4.9 \times 10^{-7}$

Table S6: Top ten NHGIS subdivisions by posterior-mean fatalities per capita per decade.

**Model**	**Decade**	n	**FFL WAIC**	**FS/S WAIC**	**ΔWAIC (FS/S − FFL)**
**High/Low Arm** (focal)	all	463	-1144.05 ± 0.28	-1170.98 ± 0.29	-26.93 ± 0.40
	1980s	8	-62.01 ± 7.58	-62.66 ± 7.01	-0.65 ± 10.32
	1990s	98	-182.88 ± 0.09	-180.52 ± 0.09	$+2.36 \pm 0.13$
	2000s	101	-227.69 ± 0.11	-244.05 ± 0.13	-16.36 ± 0.17
	2010s	140	-441.84 ± 0.22	-450.03 ± 0.22	-8.19 ± 0.31
	2020s	116	-229.63 ± 0.11	-233.73 ± 0.11	-4.09 ± 0.16
**+ Max-Select**	all	463	-1146.71 ± 0.28	-1174.48 ± 0.29	-27.77 ± 0.40
	1980s	8	-61.86 ± 7.43	-62.04 ± 7.04	-0.18 ± 10.24
	1990s	98	-183.69 ± 0.09	-183.19 ± 0.09	$+0.50 \pm 0.13$
	2000s	101	-227.81 ± 0.11	-241.96 ± 0.13	-14.15 ± 0.17
	2010s	140	-443.29 ± 0.22	-451.85 ± 0.22	-8.56 ± 0.31
	2020s	116	-230.07 ± 0.11	-235.44 ± 0.11	-5.38 ± 0.16
**Baseline ABM**	all	463	-1178.48 ± 0.28	-1197.30 ± 0.29	-18.83 ± 0.41
	1980s	8	-63.04 ± 6.26	-62.95 ± 6.14	$+0.09 \pm 8.77$
	1990s	98	-185.82 ± 0.09	-185.18 ± 0.09	$+0.64 \pm 0.13$
	2000s	101	-231.25 ± 0.11	-248.57 ± 0.13	-17.33 ± 0.17
	2010s	140	-458.22 ± 0.22	-459.66 ± 0.22	-1.44 ± 0.31
	2020s	116	-240.15 ± 0.12	-240.94 ± 0.12	-0.79 ± 0.17

Table S7: Decade-decomposed WAIC comparison of the FFL-density proxy and the FS/S longitudinal proxy across the three principal ABM families. Cell entries are WAIC ± bootstrap SE; ΔWAIC is signed so that negative values indicate that FFL outperforms FS/S. The full-record ranking of model families is identical under both proxies (High/Low Arm > Max-Select > Baseline ABM), and FFL outperforms FS/S overall for every family despite FS/S's longitudinal coverage advantage.
